# Performance of Google’s Artificial Intelligence Chatbot “Bard” (Now “Gemini”) on Ophthalmology Board Exam Practice Questions

**DOI:** 10.7759/cureus.57348

**Published:** 2024-03-31

**Authors:** Monica Botross, Seyed Omid Mohammadi, Kendall Montgomery, Courtney Crawford

**Affiliations:** 1 Ophthalmology, Burnett School of Medicine at Texas Christian University, Fort Worth, USA

**Keywords:** llm, gemini, ophthalmology, american board of ophthalmology, wqe, okap, artificial intelligence, medical education, chat gpt, bard

## Abstract

Purpose: To assess the performance of “Bard,” one of ChatGPT’s competitors, in answering practice questions for the ophthalmology board certification exam.

Methods: In December 2023, 250 multiple-choice questions from the “BoardVitals” ophthalmology exam question bank were randomly selected and entered into Bard to assess the artificial intelligence chatbot’s ability to comprehend, process, and answer complex scientific and clinical ophthalmic questions. A random mix of text-only and image-and-text questions were selected from 10 subsections. Each subsection included 25 questions. The percentage of correct responses was calculated per section, and an overall assessment score was determined.

Results: On average, Bard answered 62.4% (156/250) of questions correctly. The worst performance was 24% (6/25) on the topic of “Retina and Vitreous,” and the best performance was on “Oculoplastics,” with a score of 84% (21/25). While the majority of questions were entered with minimal difficulty, not all questions could be processed by Bard. This was particularly an issue for questions that included human images and multiple visual files. Some vignette-style questions were also not understood by Bard and were therefore omitted. Future investigations will focus on having more questions per subsection to increase available data points.

Conclusions: While Bard answered the majority of questions correctly and is capable of analyzing vast amounts of medical data, it ultimately lacks the holistic understanding and experience-informed knowledge of an ophthalmologist. An ophthalmologist’s ability to synthesize diverse pieces of information and draw from clinical experience to answer complex standardized board questions is at present irreplaceable, and artificial intelligence, in its current form, can be employed as a valuable tool for supplementing clinicians' study methods.

## Introduction

The emergence of artificial intelligence (AI) chatbots in recent years has markedly changed the landscape of education and access to knowledge. Chatbots have been demonstrated to be highly adept at synthesizing information from a wide variety of sources, effectively sorting through thousands of data points in order to generate relevant and accurate answers. The role of these large language models (LLMs) in medicine is still relatively unclear due to their novelty. The question of whether such systems can serve as substitutes for the expansive knowledge and experience of trained physicians is highly debated [1]. Despite the apparent limitation of a lack of experience-informed medical decision-making ability, which distinguishes physicians and enables them to make complex diagnoses, there has been a heightened interest in assessing chatbots’ performance on medical examinations routinely administered to physicians. LLMs such as Bard (owned by Google, based in Mountain View, California) and ChatGPT (owned by Open AI LP, based in San Francisco, California) can be trained with exposure to large volumes of training data. This can then be used to teach the LLMs appropriate patterns and relationships between data to be able to make correct predictions. Because outputs are based on provided training data, LLMs can fine-tune their knowledge with increased data exposure to smaller and more specific data subsets, enabling them to refine and strengthen their understanding of a particular domain [2].

A significant component of medical education in the United States involves rigorous standardized multiple-choice examinations designed to test trainees’ knowledge base, critical thinking skills, and clinical acumen uniformly and systematically. The examinations are typically vignette-based, testing a myriad of clinical problems and diagnostic questions likely to be encountered in a real-world setting. An ophthalmologist in the United States must pass all three parts of the United States Medical Licensing Examination, the in-service Ophthalmic Knowledge Assessment Program (OKAP) examination administered each year of residency, and the written qualifying exam (WQE) administered by the Board of Ophthalmology. Question banks are among the most popular tools used by trainees to prepare for these exams, as they often reflect similar content and formatting. Popular question banks include UWorld, OphthoQuestions, StatPearls, and BoardVitals, among others. 

Various studies have examined the performance of the most popular chatbot, ChatGPT, in various medical licensing and certification exams. Fewer have assessed the performance of Bard, Google’s AI chatbot, in similar examinations. Now “Gemini” as of December 2023, Bard was first released in March of 2023 and functions similarly to its more famous counterpart, ChatGPT, with the primary difference being the data source. While Bard continually draws from the internet to obtain the latest information, ChatGPT only connects to the internet in its premium version, and otherwise, its database is limited to sources prior to September 2021 [3]. Bard’s functions are various and include translating languages, summarizing texts and articles, solving mathematical problems, creative writing, and answering complex questions in dozens of fields through systematic retrieval of information. The performance of the LLM has been evaluated in a few different specialty assessments, including the neurosurgery oral board preparation question bank [4], the plastic surgery in-service intern year examination [5], and the nephrology board renewal self-assessment [6]. To our knowledge, however, Bard’s performance on the ophthalmology boards and board-style questions has never been evaluated. This study aims to assess Bard's performance in answering practice questions for the ophthalmology board certification exam. We hypothesize that the chatbot will be able to answer the majority of questions presented correctly. The results of this study would add to the existing literature on Bard's competency in answering complex standardized medical questions in yet another specialty. Future discussions would focus on the utility of Bard as a study adjunct for ophthalmology trainees preparing for certification exams and its innovative role in enhancing medical education in a fast-advancing technological landscape. 

## Materials and methods

Two hundred and fifty multiple-choice questions from the “BoardVitals” ophthalmology exam question bank (founded in 2012 by Andrea Paul, MD and Daniel Lambert) were randomly selected and entered into Bard to assess the AI chatbot’s ability to comprehend, process, and answer complex scientific and clinical ophthalmic questions. A random mix of text-only and image-and-text questions were selected from 10 subsections. The subsections tested were basic sciences, pediatric ophthalmology, cataract, cornea and external disease, glaucoma, neuro-ophthalmology, uveitis, retina and vitreous, ocular oncology, and oculoplastics. Each subsection included 25 questions. Around a quarter of the questions entered into Bard were not understood by the LLM and were therefore omitted from the total score. The percentage of correct responses was calculated per section, and an overall assessment score was determined.

## Results

On average, Bard answered 62.4% (156/250) of questions correctly. The worst performance was 24% (6/25) on the topic of “Retina and Vitreous,” and the best performance was on “Oculoplastics,” with a score of 84% (21/25) (Table 1). While the majority of questions were inputted with minimal difficulty, about a quarter of the questions entered were unable to be processed by Bard, particularly questions that included human images and multiple visual files. Some of the lengthier vignette-style questions were also not understood by Bard (communicated by the LLM simply stating this rather than producing an answer) and were therefore omitted. It was determined that Bard had the greatest success with text-based questions that didn’t involve the interpretation of images that included human features or the processing of more than one visual file. 

**Table 1:** Score distribution as divided by subsection and total assessment score

Subsection	Percent Correct
Basic Science	68% (17/25)
Cataract	56% (14/25)
Cornea and External Disease	76% (19/25)
Glaucoma	76% (19/25)
Neuro-ophthalmology	64% (16/25)
Oculoplastics	84% (21/25)
Ocular Oncology	40% (10/25)
Pediatric Ophthalmology	72% (18/25)
Retina and Vitreous	24% (6/25)
Uveitis	64% (16/25)
	Total Score: 62.4% (156/250)

## Discussion

The results of this study underscore the competence of chatbots in answering complex medical and diagnostic questions designed to test physicians' knowledge in training. Models like Bard have the potential to be an adjunct tool for ophthalmologists in training: Bard is accessible and convenient, allowing flexibility that is beneficial to busy medical professionals and may aid traditional study methods. AI-powered chatbots may help learners personalize their approach to studying by tailoring it to their individual needs or helping identify areas of weakness and can offer the benefit of immediate feedback [7].

While Bard answered 62.4% (156/250) of questions correctly and is capable of analyzing vast amounts of medical data, it ultimately lacks the holistic understanding and clinical experience of an ophthalmologist. Despite advancements in natural language processing, chatbots like Bard may struggle to comprehend the full context of complex medical concepts and scenarios. This limitation can lead to inaccuracies or misunderstandings in responses, potentially undermining the educational value of the interaction or ultimately having real-world consequences that may result in harm to patients due to misdiagnosis or incorrect choice of treatment. 

A similar study published in January 2024 by Haddad and Saade [8] found that ChatGPT answered 46.77% (n=116) of ophthalmology board review questions correctly with the 3.5 model and 62.90% (n=156) of questions correctly with the 4.0 version. Although this study used a different source for practice questions (from the book Ophthalmology Board Review Q&A), these results show that Bard performs similarly to ChatGPT 4.0 on ophthalmology board-style review questions. A study from October 2023 that examined the ability of other LLMs to answer ophthalmology board-style questions determined that other models had difficulty answering multi-step inference-type questions and performed better overall on single-step deductive reasoning questions [9]. To pass the American Board of Ophthalmology’s WQE, a test-taker must achieve a scaled-cut score of 700 [10]. The raw, percent-correct score differs each year due to the nature of the scaled transformation, but the percent correct to pass was estimated to be 60% in 2018 and 65% in 2019 [11]. Taking into account the results from our study, Haddad and Saade’s study, and these estimated percent-correct passing values, both Bard and ChatGPT 4.0 have the potential to score at or near passing on the WQE.

Over-reliance on AI-driven tools may hinder critical thinking skills and independent problem-solving abilities that are necessary for clinical practice. There is a risk that learners may increasingly depend on chatbots for studying and neglect other essential learning resources and strategies. The reality is that real-world patients and clinical scenarios almost always involve layers and depth of knowledge, require multi-step inferences, and demand clinicians to tailor their approach to an individual rather than rely on broad categories or assumptions. Therefore, although Bard may have the potential to pass ophthalmology board exams, it is unlikely that, in the current state of its technology, it is approaching the capability of successfully substituting the clinical decision-making abilities of a board-certified ophthalmologist. This may evolve over the coming years with enhanced training of the LLM and increased exposure to the latest data in the field. Google’s latest version of Bard, Gemini, was released in December 2023 (shortly after the completion of this study) and is said to be trained in a larger and more diverse dataset. This could potentially mean that it may have an enhanced ability to understand and answer the questions asked of Bard throughout this study. Future investigations will focus on testing Gemini’s performance and comparing it to that of its preceding version in order to assess whether increased training and a wider available dataset would translate to a higher test score. 

Other limitations of Bard’s ability to take and pass board-style ophthalmology questions include its inability to analyze complex images reliably. Much of clinical practice in ophthalmology depends on the physician’s competency in correctly interpreting images on slit-lamp examination, diagnostic testing modalities, or other visual inspections of the eye. Bard’s inability to reliably assess questions with complex visual components and multiple image files indicates a significant shortcoming. Its failure to interpret questions containing images with human features, such as exterior eyelid lesions and other extraocular pathologies, is also noteworthy. Consequently, our final percent correct may be inflated and overestimating Bard’s testing capabilities, given questions that could not be understood were excluded from the total.

Furthermore, the setbacks of AI chatbots extend beyond their cognitive abilities. These systems rely heavily on the data they are trained on, and their performance is only as good as the data available to them. In the case of medical chatbots, access to comprehensive and up-to-date medical literature and clinical guidelines is crucial for accurate decision-making. However, constraints such as paywalls and restricted access to proprietary databases can hinder the chatbots' ability to access the most relevant and current information. At the time that this research was conducted, when asked if it could cite up-to-date information from recently published scientific journal articles, the chatbot stated that much of the most recent data and access to full articles are blocked by paywalls and other restrictive measures, limiting its ability to pull information from the most accurate, reliable, and current sources. This limitation not only affects the chatbot's performance but also raises concerns about the quality and reliability of the information it provides to users, potentially compromising patient safety. The development of AI chatbots for healthcare applications must also contend with ethical considerations and patient privacy concerns. As these systems become more integrated into clinical practice, ensuring patient confidentiality and data security becomes paramount. The potential risks associated with breaches of patient privacy underscore the need for robust regulatory frameworks and ethical guidelines to govern the development and deployment of AI-driven healthcare solutions. 

Limitations of this study include the inability to extrapolate between board-style practice questions and actual board examination questions. We did not assess how closely BoardVitals questions reflect real OKAP or WQE content, and therefore, in reality, Bard’s performance on these exams may not be accurately represented in our data. Additionally, there are presumed limitations in the reproducibility of our results. AI-driven chatbots like Bard are designed to generate unique answers each time a prompt is given, and therefore, repeated attempts at asking board-style ophthalmology questions could yield different results.

AI technology, including LLMs like Bard, is a quickly expanding field of applied science that has the potential to benefit medicine greatly. User feedback and continued technological improvement of these models allow for rapid development and advancement in the field of AI-driven chatbots, and so outcomes from studies like this are only projected to improve.

## Conclusions

This study aimed to evaluate the performance of Google’s AI chatbot Bard on ophthalmology board exam practice questions. The study's findings highlight the indispensable clinical judgment, nuanced diagnostic ability, and experience-informed medical decision-making of a board-certified ophthalmologist. While it is likely that chatbots will continue to improve as the landscape of AI advances, it is unlikely that, in their present state, they are at a point of substituting trained physicians. This study adds to the existing literature investigating Bard’s performance on standardized medical licensing and board examinations. Future studies will examine the performance of Bard’s newer version, Gemini, which was released shortly after the completion of the study, to assess whether chatbot training on larger and more diverse datasets would translate to improved performance.
